# Parsimonious test of dynamic interaction

**DOI:** 10.1002/ece3.4805

**Published:** 2019-02-08

**Authors:** Sarah Chisholm, Andrew B. Stein, Neil R. Jordan, Tatjana M. Hubel, John Shawe‐Taylor, Tom Fearn, J. Weldon McNutt, Alan M. Wilson, Stephen Hailes

**Affiliations:** ^1^ Computational Statistics and Machine Learning University College London London UK; ^2^ Department of Computer Science University College London London UK; ^3^ University of Massachusetts Amherst Amherst Massachusetts; ^4^ Botswana Predator Conservation Trust Maun Botswana; ^5^ Landmark College Putney Vermont; ^6^ School of Biological, Earth and Environmental Sciences Centre for Ecosystem Science University of New South Wales (UNSW) Sydney New South Wales Australia; ^7^ Taronga Conservation Society Australia Taronga Western Plains Zoo Dubbo New South Wales Australia; ^8^ Structure & Motion Laboratory The Royal Veterinary College Herts UK; ^9^ Department of Statistical Sciences University College London London UK

**Keywords:** African wild dogs, analysis, association, avoidance theory, ecology, GPS, leopards, permutations, statistics

## Abstract

In recent years, there have been significant advances in the technology used to collect data on the movement and activity patterns of humans and animals. GPS units, which form the primary source of location data, have become cheaper, more accurate, lighter and less power‐hungry, and their accuracy has been further improved with the addition of inertial measurement units. The consequence is a glut of geospatial time series data, recorded at rates that range from one position fix every several hours (to maximize system lifetime) to ten fixes per second (in high dynamic situations). Since data of this quality and volume have only recently become available, the analytical methods to extract behavioral information from raw position data are at an early stage of development. An instance of this lies in the analysis of animal movement patterns. When investigating solitary animals, the timing and location of instances of avoidance and association are important behavioral markers. In this paper, a novel analytical method to detect avoidance and association between individuals is proposed; unlike existing methods, assumptions about the shape of the territories or the nature of individual movement are not needed. Simulations demonstrate that false positives (type I error) are rare (1%–3%), which means that the test rarely suggests that there is an association if there is none.

## 1 INTRODUCTION

Methods for collecting data on the movement of animals have advanced dramatically over the last two decades, with GPS and inertial measurement units becoming smaller, lighter, more energy efficient, and more accurate than ever before. These developments enable the detailed tracking of multiple individuals over long periods of time. To make the most of these technological advances, methods to analyze large amounts of data efficiently are essential.

The interactions of animals are one area of application in which vast amounts of data are collected; yet, efficient methods to analyze them are scarce. When analysing the interaction between solitary animals, the quantification of association or avoidance between territorial conspecifics would advance our understanding of animal ecology and, in the long term, the impact of changing environments. Existing forms of such tests are predicated on assumptions about the shape of each individual's territory (Dunn, 1979; Macdonald, Ball, & Hough, 1980) or, more recently, model the animal's movements as a random walk (Fortin et al., 2005; Latombe, Parrott, Basille, & Fortin, 2014; Potts, Mokross, Stouffer, & Lewis, 2014; Vanak et al., 2013). Perhaps for that reason, these methods are often not employed, and avoidance is instead inferred from circumstantial evidence. For example, Jackson and Ahlborn comment in (1989) that “judging by the intensity of use of core areas, the large amount of overlap among individuals, and the relatively small total home areas, it is remarkable that the tagged cats managed to remain on average >2 km apart. This implies that the Langu cats [snow leopards (*Panthera uncia*, Schreber, 1775)] actively avoided one another, while sharing the same area.” There is no explanation as to why an average of 2 km could not have occurred purely by chance; rather, an absence of contacts is seen as evidence of active avoidance.

A test for dynamic interactions was first suggested by Macdonald et al. (1980); this is based on the application of a quadrivariate normal distribution to the co‐ordinates of the two target individuals. Dunn describes a similar approach that employs a multivariate Ornstein‐Uhlenbeck model rather than a multivariate normal model (Dunn, 1979). Sunarto, Kelly, Parakkasi, and Hutajulu (2015) use kernel density estimation (KDE) to characterize activity patterns for each species and calculate the coefficient of overlap between pairs of wild cat species. These tests either require that the utilization of each range is distributed about a single center of activity or in an oval shape. Violation of these assumptions, which have no obvious biological basis, can produce large errors (Doncaster, 1990).

Delgado, Penteriani, Morales, Gurarie, and Ovaskainen (2014) proposed a functional response in which social behavior is assumed to depend on proximity to other individuals. As detailed by the authors, the null model is supposed to account for all factors influencing movement behavior apart from conspecifics. In their method, they suggest a null model that is calculated from movement in a random direction with the same step length as the observed movement. Similarly, Fortin et al. (2005) proposed a method that compares characteristics of the observed movements to characteristics based on a correlated random walk. This was later used to test for interactions by Latombe et al. (2014), Potts et al. (2014), Vanak et al. (2013), and others (Merkle, Fortin, & Morales, 2014; Thurfjell, Ciuti, & Boyce, 2014). This method assumes that the individuals would move randomly if they were not directly reacting to another individual or environmental factors. This means that specific habitat areas with higher or lower chances of being visited have to be specifically incorporated into the null model. As an example, a particularly dense area of the habitat might be difficult to penetrate or represent an area with few possibilities for hunting. If these areas are not included in the model they could increase false positive results because the ranges of the focal individual and their conspecifics might be organized so that the individuals are limited to moving in regions that cause the observed distances between them to be smaller than expected by chance.

Elbroch, Quigley, and Caragiulo (2014) suggested a generalized linear model to test for predictive power of various factors on the number of spatial associations observed. These factors included the number of elk in the study area and the mean genetic relatedness between interacting individuals. This is an interesting approach that helps in understanding what factors influence associations; however, it does not easily extend to testing whether individuals actively avoid each other or seek each other's proximity.

Doncaster suggested the first non‐parametric test in Doncaster (1990). This compares the empirical distribution function of the *N* paired separations with that of the complete set of *N* ^2^ separations. For this, a critical separation is chosen, within which the presence of interactions is deemed to be interesting. However, the correct value of this separation may not be easy to estimate and the number of observations would have to be very large to permit an analysis over multiple different separations. Furthermore, the significance test depends on the independence of successive data points and is only valid for fixed ranges of inter‐individual separation (Doncaster, 1990).

In this paper, we propose a method that creates perturbations of blocks (e.g., days) of the observed data as a null model. It is therefore possible to create up to *D*! (where ! stands for the factorial and *D* is the number of blocks in the observation period) permutations to which to compare the observed data; there is no need to assume independence between individual measurements, only between blocks of measurements (e.g., days or weeks). One of the main advantages of this method is that specific geographic areas that are visited less or more frequently by the individuals do not have to be included manually. Instead, they are automatically accounted for, since the frequency with which each location is visited remains exactly the same in both the null model (the permutations) and the observed movement. A further benefit is that there is no need to guess in advance which range of separation distances might constitute an interaction; rather, one just applies the test of interaction over multiple different distance ranges.

## 2 METHOD

Dynamic interactions can be measured in two ways, as defined by Doncaster (1990). The first is termed “static interaction”, which describes a spatial overlap of home ranges, as is discussed for example in Benhamou, Valeix, Chamaill‐Jammes, Macdonald, and Loveridge (2014) and Ngoprasert et al. (2012). The second characterizes dependencies between individuals’ movements. This study examines the latter, “dynamic interactions”. As Doncaster describes in (1990): “Dependency in the movements of two individuals (dynamic interaction) […] can be expressed in terms of probability. Are the animals more likely to maintain a certain separation (positive dynamic interaction) or less likely (negative dynamic interaction) than is expected from the configuration and utilization of their ranges? At small separations in particular, does there exist a bond of attraction between them or do they respond to close contact by mutual repulsion?”

The method described in this paper does not assume any underlying distribution, nor a particular shape or usage of the individuals’ territories. It does not require independence of consecutive measurements, nor a constant time difference between the measurements. This test simply relies on the disassociation of the target individuals by using permutations. To accomplish this, the observation period is divided into time blocks, such as days. These blocks are then permuted for each animal individually and distances calculated; consequently, the inter‐individual distances at, say, 2 p.m. will be calculated from the locations of the animals on different days at 2 p.m. Assuming that the blocks are independent, this approach can be used to obtain the inter‐individual distances that one would expect to see if the animals did not respond to each other's whereabouts.

Following is a detailed description of the steps taken to test for association or avoidance using the proposed method.^1^

First, the distance between two individuals is calculated at each point in time using the observed data. Where data points are missing or observations are taken at different times, the positions are interpolated linearly, for simplicity, as has been done previously (Fortin et al., 2005; Turchin, 1998). The observed data are then divided into blocks which are deemed to be independent; if these are days or weeks then the diel/weekly movement patterns remain intact in the permutations (for example, a propensity to visit a waterhole at 8 a.m. or sleep at 12 p.m.). These blocks are permuted randomly 10,000 times and the distance between the target individuals at each time point is recalculated for each permutation.

There are now one observed and 10,000 permuted lists of inter‐individual distance measurements for each time point in the data. It is, consequently, possible to determine how likely the observed measurements are, given the permuted set that is our null model. If individuals are significantly more often found close together in the observed data set than in the permuted data sets, then one can conclude that this is unlikely to have arisen by chance. Likewise if they are significantly less often seen together.

It remains to be determined what “close together” means in this context. It would, for example, be reasonable to choose a range of distances, say 0–20 m to represent the region in which physical contact is most likely; alternatively, since animals communicate explicitly by sound and implicitly by sight, one might be interested in other ranges—say 80–100 m. Additionally, visibility will vary across habitats. As discussed above, one of the advantages of this approach is that it is possible simultaneously to test for interesting interactions (or the lack of them) across a set of ranges. Thus, in Section 3.2.1, which describes the application of this method to data collected from leopards (*Panthera pardus*, Linnaeus, 1758), the range intervals are chosen to be 0–20, 20–40, 40–80, 80–160, 160–320, and 320–640 m. Whatever set is chosen, the separation distances calculated from the observed and permuted data are binned according to the selected set of intervals; this gives us a count of the number of times that the individuals were in each distance range for both the observed and permuted data sets.

The null hypothesis is that there is no difference between the number of times the target individuals are found within a certain distance interval in the observed and permuted time series. The two alternatives are that the individuals are (a) more often; and (b) less often in the interval examined than expected from the permutations.

A *p*‐value is defined to be the probability of obtaining a result at least as extreme as the one that was observed, assuming that the null hypothesis is true (Goodman, 1999). Therefore the *p*‐value in this case is the upper bound (as we only have a sample of all possible scenarios) on the proportion of permutations as extreme, or more extreme, than the observation. Say the two target individuals were observed to be in the 20–40 m interval *M* times, then the *p*‐value for the null hypothesis versus the alternative that the individuals are less often in the same interval than expected by chance lies between:

$$\frac{n_{1}}{n_{\mathrm{perm}}}\leq p<\frac{n_{1}+1}{n_{\mathrm{perm}}}$$

where $n_{\mathrm{perm}}$ is the number of permutations calculated and $n_{1}$ is the number of permutations in which the dyad was inside the 20–40 m interval at most *M* times. The observed number of times the individuals were within that particular interval is then compared to the distribution created by the permutations. Observations lying in the 0.05/2*k* tail of the permutations will be regarded as evidence that the target individuals were less often in the distance interval than expected by chance. This percentage is calculated using the Bonferroni correction (Morrison, 1990); the 0.05 represents the significance level, which has to be divided by 2*k*, where *k* is the number of distances tested, since we test *k* distance intervals for avoidance and *k* for association. The Bonferroni inequality balances out the effect of multiple testing.

A *p*‐value for a given distance interval that is less than 0.05/2*k* indicates that there is strong evidence against the null‐hypothesis that the two individuals are within that interval as often as expected by chance. This could be because they are more often within that particular distance interval, as would be the case if they actively sought each other out, or because they are less often within the interval, which would suggest active avoidance.

### 2.1 Simulations

The method proposed in this paper is focused on determining whether a pair of individuals (or social groups that they are members of) have been more or less in contact than expected. To show the accuracy of the method, neighboring leopard movements were simulated and spatio‐temporal associations were imposed on some of those movement patterns. The test was applied to these simulated movements and the proportion of correctly identified associations and non‐associations was calculated.

**Figure 1:**
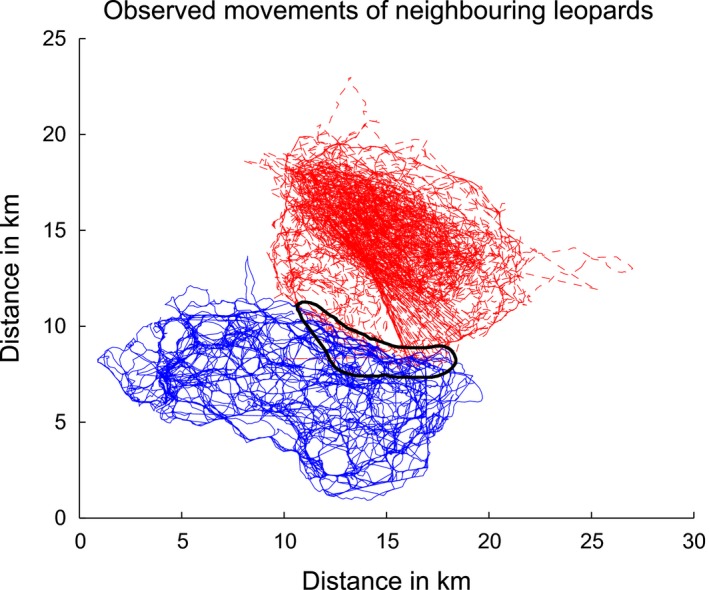
Movement of the individuals used for approximate territory size/shape/overlap and step size distribution. The overlapped area is circled by a black line. The observation period is 217 days

Movements were simulated using simple random walk processes. These processes are defined by an equal probability of moving in any direction at each step (the direction is chosen from a uniform distribution on the range from 0 to 2*π*). The shape of the territory was assumed to be elliptical with radii of 9 and 4 km. These parameters are roughly estimated from the observations collected on one of the leopards (randomly selected) used in Section 3.2.1. Figure 1 shows the areas visited by this individual (blue solid line) and its neighbor (red dashed line) during the observation period.

**Figure 2:**
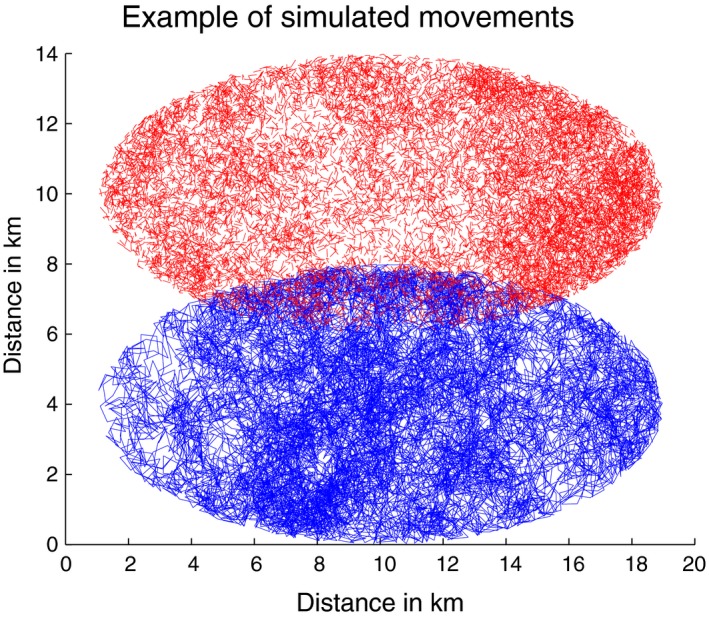
Representative example of the simulations. The observation period for this simulation example is 350 days, the association time is three steps, and the sensing distance is 300 m

The territories of both the simulated movements have the same size and shape. The second territory is shifted by 6 km along the axis of the minor radius. Therefore the overlap is similar to that of the observed individuals. The overlap was overestimated as false positives are more readily detectable if the likelihood of encounters is increased. An example of the simulations is given in Figure 2. It is clear that the simulations are very different to the observed movement patterns; however, mimicking the movement of the leopards is not the aim of these simulations. The method should be capable of finding associations since the precise nature of movement is not critical to its operation, as long as there are no major changes in the movement during the observation period.

Ten thousand eighty simulations were run without any association between the dyad and a further 10,080 simulations were run in which the individuals actively seek each other's proximity if they are within a certain distance of each other (from here on referred to as the *sensing distance*). In the simulations in which there is no association between the individuals, both processes are simple random walks with elliptical boundaries. In the cases with association, the general movement is again a simple random walk except when they are within the sensing distance of each other. In that case, they move directly toward each other and stay at one unit less than half the sensing distance from each other (this will be referred to as the *association distance*). The individuals stay within that distance of each other between 1 and 5 steps (this number of steps will be referred to as the *association time*).

Within the 10,080 simulations with associations, the association time is varied from 1 to 5 steps and the sensing distance is varied from 250 to 500 m. For both the simulations with association and the simulations without association, the observation period is varied from 100 to 350 days. For each of the simulations, the method proposed in this paper is applied, and it is recorded whether the method correctly detects that there is, or is not, an association.

For each simulation, four distance intervals are tested for associations. In the association scenario, one of these intervals contains the association distance and should therefore test positive for a more than expected (MTE) association, that is, the individuals are more often within those distances than expected by chance. As a corollary, the interval containing the association distance should test negative for a less than expected (LTE) association. The other three intervals tested are outside the sensing distance and should therefore test negative for both MTE and LTE. In the scenario without association, all of the intervals should test negative for both MTE and LTE.

In concrete terms, the total number of distances tested for an association in each of the two scenarios is 40,320 (=4 × 10,080). When there is no association, all 40,320 distances should test negative for MTE (MTEneg) and LTE (LTEneg) with no positive MTE (MTEpos) or LTE (LTEpos) tests. In the association scenario, 10,080 should be MTEpos and LTEneg, representing situations in which they are correctly identified as being more often (not less often) within the interval than expected when the interval contains the association distance. In a similar fashion, 30,240 should be MTEneg and LTEneg. In total, this gives expected values of 30,240 for MTEneg, 40,320 for LTEneg, zero LTEpos and at most 10,080 for MTEpos. The latter is slightly complicated by the fact that animals only seek each other's proximity if they happen to be within the sensing distance of each other; this will not occur in all simulations.

### 2.2 Application

To demonstrate how the method could be used, the proposed method has been applied to location data collected from eight resident neighboring leopards and eight packs of African wild dogs (*Lycaon pictus*, Temminck, 1820) in Northern Botswana. In the former case, each of the leopards was fitted with a GPS collar and the aim of the test was to identify whether the individual leopards avoided each other. For African wild dogs, one individual in each pack was fitted with a GPS collar. The purpose in this case was to determine whether neighboring packs avoided each other (or alternatively sought proximity).

#### 2.2.1 Leopards

Between 2007 and 2012, two female and six male leopards were fitted with GPS collars. Not all collars were fitted for the entire study period; therefore only periods of simultaneous tagging were used in this analysis. The number of days each dyad was simultaneously tagged varied between 119 and 406. Locations were measured at least four times a day. As leopards are generally active at night and are least active in the middle of the day (Bailey, 1993) a day was considered to run from midday to midday for the purposes of permutation. The distance intervals tested were: 0–20, 20–40, 40–80, 80–160, 160–320, and 320–640 m. Bins at short distances were chosen narrower than distances further apart, because we were particularly interested in close proximity of the individuals.

#### 2.2.2 African wild dogs

The eight packs of African wild dogs were collared between May 2011 and May 2014. As for the leopards, the wild dog packs were not all collared simultaneously. Therefore, packs were only considered if they were tagged simultaneously for at least 100 days. The resulting 14 neighboring pack dyads were collared for between 108 and 402 days. The distance intervals tested were: 0–500 m, 500 m–1 km, 1–1.5 km, and 1.5–2 km.

## 3 RESULTS

In the following two sections, the results of the simulations are detailed, followed by the results from the application. First, the results of the simulations in which there is an association are described in Section 3.1.1, then those with no association (Section 3.1.2). This is followed by the results of the application to leopard data (Section 3.2.1) and to African wild dog data (Section 3.2.2).

### 3.1 Simulations

#### 3.1.1 Association scenario

This section discusses the results in the scenario in which there is an association between the two simulated individuals. Overall, out of the 40,320 expected LTEneg intervals tested, 37,940 (94%) were correctly identified as not being less often within close proximity of each other than expected by chance. Out of the 30,240 expected MTEneg intervals, 29,761 (98%) were correctly identified as not being more often within close proximity of each other. And, out of the 8,975 MTEpos intervals that are possible (these are the only occasions on which the individuals end up within the sensing distance of each other by chance, as discussed in Section 2.1) 7,250 (81%) were correctly identified as being more often within close proximity of each other than expected.

**Figure 3:**
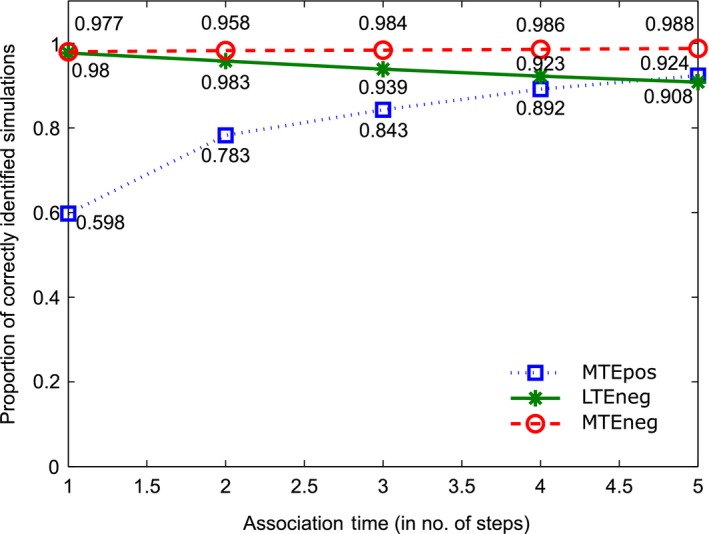
Proportion of simulations correctly classified as not having a less than expected association (LTEneg, i.e., “no avoidance”), not having a more than expected association (MTEneg, i.e., “no attraction”) and having a more than expected association (MTEpos, “attraction”) as a function of the number of time steps spent at the association distance

Figure 3 shows the results broken down by the association time, that is, by how many steps the individuals stay within the association distance of each other, before they go back to a random walk. The results are detailed in Supporting Information Table S1, with the number of simulations and correctly identified distances listed.

The LTEneg and MTEneg results are consistently highly accurate, with false positives between 1% and 9% of cases. This demonstrates, that the test very rarely suggests that there is an association, when there is none. The LTEneg and MTEneg results should not be affected by the time spent within the association distance, since they check the distances that are outside the association distances. This can be seen in the results, as the LTEneg and MTEneg lines are close to horizontal.

The general upward trend of the MTEpos line is to be expected. The more time the individuals spend within a distance interval, the higher the likelihood of the test detecting an association. The proportion of correctly identified intervals increases from 60% when only one time step was spent within the association distance, to 92% when five time steps were spent within the association distance.

**Figure 4:**
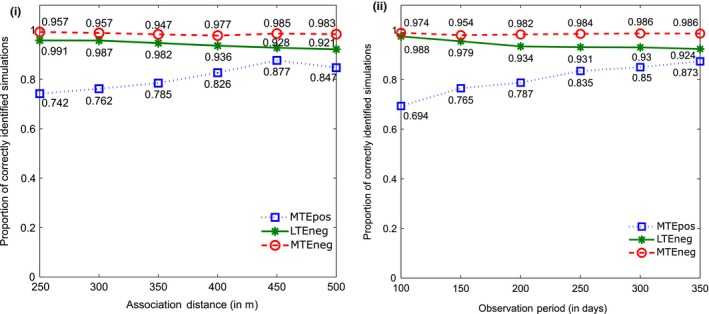
Proportion of simulations correctly classified as having a LTEneg, MTEneg, and MTEpos association as a function of (a) the size of the association distance and (b) the length of the observation period

The breakdown of the results with respect to the varying sizes of the association distance and lengths of observation period are presented in Figure 4a,b respectively. The absolute number of simulations and number of correctly identified simulations are listed in Supporting Information Tables S2 and S3.

The results are very similar to those characterized by the association time. False positive results (MTEneg and LTEneg) were suggested between 1% and 8% of the cases. The MTEpos results show that with a larger association distance, or with a longer observation period, the test is more likely to detect an association correctly. This is probably the case, because the individuals are more likely to be within the sensing distance of each other during the simulations and therefore show an association more often than in the cases in which the association distance is small or the observation period is short.

#### 3.1.2 No association scenario

This section discusses the results in the scenario in which there is no association between the two individuals. In this case, all 40,320 distances tested should indicate that the individuals were not less often than expected within the distance intervals tested (LTEneg) and neither were they more often than expected within those intervals (MTEneg).

Overall, out of the 40,320 LTEneg tests 39,915 (99%) were correctly identified as not being less often than expected within the intervals tested. And out of the 40,320 MTEneg tests 39,455 (98%) were correctly identified as not being more often than expected within those intervals.

**Figure 5:**
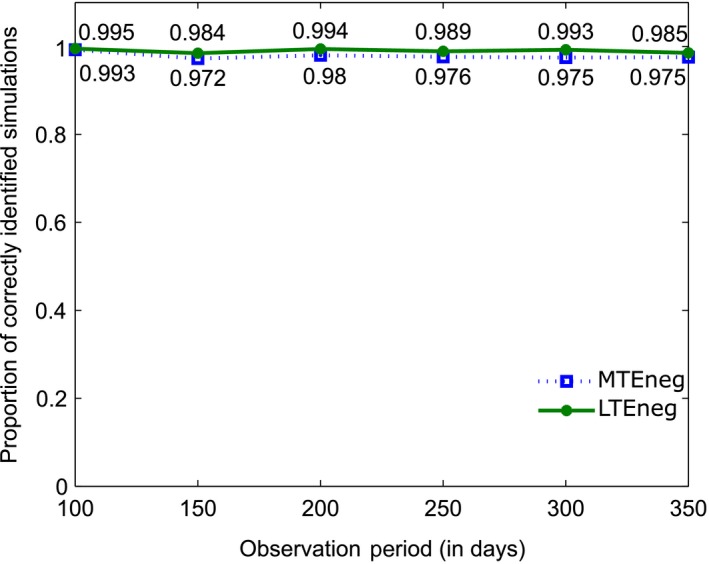
Proportion of simulations correctly classified as having a LTEneg and MTEneg association as a function of the length of the observation period

The break down of the results with respect to the length of observation period is presented in Figure 5 and detailed, including absolute number of distances tested and number of correctly identified significant intervals, Supporting Information Table S4. The results are consistently high, with accuracies between 97% and 99%. This suggests that the method has a small type I error (false positive) of between 1% and 3%.

In summary, the results demonstrate that false positives (type I error) are rare, which means that the test rarely suggests that there is an association if there is none. When there is no association, between 94% and 99% of cases are correctly identified as not having an association. The results for MTEpos, that is, the correct identification of an association, are consistently lower than the results for LTEneg and MTEneg (the correct identification of no association). We believe that a small false positive result is more important than a small false negative result, that is, suggesting that the individuals do not show an association when there is one, is favoured over suggesting that the individuals show an association when there is none.

### 3.2 Application

Location data were collected in latitude and longitude format. Before the analysis, the latitude and longitude were transformed into Cartesian coordinates using the dg2lg function from the Geodetic Toolbox in Matlab (MATLAB, 2014).

#### 3.2.1 Leopards

Applying the proposed method to leopard data suggested that none of the dyads spent less time within close proximity of each other than would be expected by chance. This observation conflicts with the conclusions of studies suggesting that male leopards dynamically avoid one another (Hornocker, 1970; Jackson & Ahlborn, 1989; Stander, Haden, Kaqece, & Ghau, 1997) to reduce the likelihood of violent or fatal conflicts (Bailey, 1993; Brown, 1982). These data demonstrate that not only do leopards not actively avoid one another, there is little pressure for them to do so as they are highly unlikely to encounter one another by chance.

As expected, two of the six male‐female dyads (F1M2 and F2M3) were significantly more often in close proximity (F1M2: 0–160 m and F2M3: 0–80 m, 160–640 m) than expected by chance. This is most likely due to courtship and mating (Bailey, 1993).

More surprisingly, two of the five male‐male dyads (M2M3 and M3M6) were highly significantly more often in close proximity of each other (both M2M3 and M3M6 in the 0–80 m interval). The individuals in both of these dyads are of similar size and weight and are in their prime (it can not be ruled out that they are related). M2 and M3 are also the only males shown to be significantly more often in close proximity of the two females. Unfortunately, M6 was only collared simultaneously with M3, so associations between M6 and the females, or M6 and M2 could not be tested.

**Figure 6:**
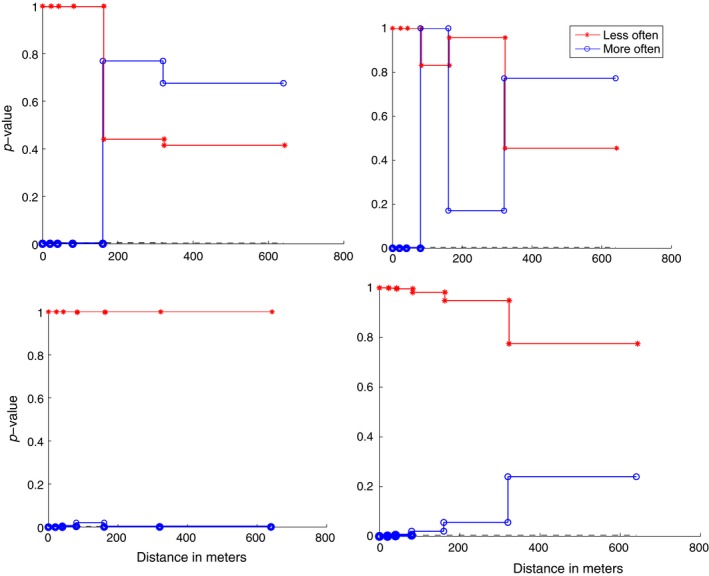
Representative *p*‐value plots of leopard avoidance and association. If the red line with stars is below 0.004 (0.05/(2*6)—the black dashed line—hardly visible here, because it is so close to the *x*‐axis) it suggests that the individuals “avoid” being within that distance of each other. If the blue line with circles is below the black dashed line it suggests that the individuals are attracted to being in that distance of each other

For each dyad, the *p*‐values per distance were plotted and the four plots belonging to F1M2, F2M3, M2M3 and M3M6 are shown in Figure 6.

#### 3.2.2 African wild dogs

**Figure 7:**
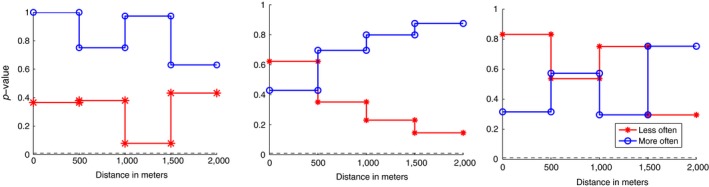
Representative *p*‐value plots of African wild dog avoidance and association. If the red line with stars is below 0.006 (0.05/(2*4)—the black dashed line) it suggests that the packs “avoid” being within that distance of each other. If the blue line with circles is below the black dashed line it suggests that the packs are attracted to being in that distance of each other

Using the proposed method on data collected on African wild dog packs suggested that none of the dyads showed any significant distance patterns, neither being less often, nor more often in close proximity to each other over the intervals considered. Three of the *p*‐value graphs, as described in Section 3.2.1, are shown in Figure 7.

## 4 DISCUSSION

As Doncaster mentions in Doncaster (1990), “A positive component is likely to arise particularly when the two animals have separate resting sites at which they regularly begin and end their cycles of activity.” The implication of Doncaster's statement is that colocation, when associated with a geographic point, is of a different nature to colocation in featureless areas because one is intrinsically more likely than the other. The technique we propose here directly discounts chance interactions of this form. Assume that two individuals meet regularly at a waterhole each morning. Since we do not disrupt the diurnal cycle in permuting days, those individuals will meet regularly at that waterhole in the permuted time series as well. Consequently, to establish that a statistically significant interaction occurred in the observed data, the number of occurrences of observed colocation would need to be very high; much higher than might be accounted for by the number that occur by chance alone. Conversely, in areas in which few meetings occur by chance, a smaller number of meetings will be considered significant.

Doncaster's comments do, however, point toward a need for care in the application of our test. If the waterhole is available only for part of the year, and permutations occur across the entire year, then the interactions could appear to be significant. Consequently, it is important to ensure that permutations occur only between days/weeks that are equivalent. For example, permuting days that have very different seasonal features may well lead to spurious results.

Such seasonal features could include seasons of drought, where for example a waterhole that both individuals generally use does not exist. Seasons of very high rain fall could also change the behavioral pattern, for example by forcing one or both of the individuals to find a different resting site. Such examples illustrate when care should be taken.

To investigate such dependencies in our case, the locations of the dyads that were significantly more often colocated were plotted (see Supporting Information Figure S1 for an example of the plots relating to the leopard data). None of the location plots showed any particular geographic location as being the source of the significant proximities.

To examine possible seasonal effects, the distances between each dyad were plotted over time (see Supporting Information Figures S2 and S3 for the leopard and African wild dog data respectively). From these graphs, it can be seen that there is no particular seasonal clustering of the small number of observed colocations.

When there are observation periods in which the location of the two observed individuals is not known in enough detail (when at least one of the two individuals’ locations is recorded less than every 6 hr), that period is excluded from the analysis. This is shown in the time series plots, Supporting Information Figures S2 and S3, by periods of missing data, such as that toward the beginning of plot M3M6 in Supporting Information Figure S2.

Since positions are interpolated when data points are missing or observations are taken at different times, this could result in missing an avoidance or association response. The two individuals may be traveling in a straight line, deviate in a hemisphere to avoid the cue of another individual, and then rejoin the original route. Depending on fix intervals, the method might not detect these interactions. However, if individuals are not located simultaneously, it is impossible to know where exactly the individuals are.

Other confounding factors, such as two individuals following a third conspecific or heterospecific that has not been fitted with a GPS collar, cannot be ruled out as possible explanations for an observed relationship. But this is simply a restatement of the truism that correlation and causation are different and that causations can generally not be tested for without a randomized experiment, which is not possible in observational studies.

In general, our results support the finding of previous work on mutual avoidance/attraction between neighboring African wild dog packs (Mills & Gorman, 1997). As previous data were acquired by VHF tracking collars, it was limited to relatively few near‐simultaneous locations of neighboring packs acquired by physically tracking the animals (Mills & Gorman, 1997). Despite significant overlap between their ranges (ca. 35%; Reich, 1981), observed packs were seen to meet very rarely; until now it has not been possible to determine whether this occurred by active avoidance or simply as a consequence of natural movement. In our study, using larger volumes of data acquired remotely using GPS radiocollars, we found no evidence of active spatial avoidance or association between neighboring packs. As can be seen from the *p*‐value plots in Figure 7, our close proximity counts could have happened by chance alone at all measured distances. Spatial interactions (though not necessarily direct interactions) at our measured scales were no more or less likely to occur than would be expected by chance. In fact, our data suggest that on only eight occasions were dyads within 600 m of one another, a reasonable distance over which visual encounters seem to occur in this species (cf Jordan et al., 2017), suggesting that direct physical encounters are rare.

Although it is not yet clear by what mechanism African wild dogs establish and maintain territories, there is strong evidence they do so based on chemical signaling using scent marks (Jackson, Weldon McNutt, & Apps, 2012; Jordan, Golabek, Apps, Gilfillan, & McNutt, 2013). It is possible that scent, which can be encountered without being simultaneously colocated, holds sufficient information to indicate the continued presence of a neighboring pack and so may reduce the frequency and cost/benefit ratio of direct encounters. It would therefore be of great interest to investigate the temporal association/avoidance in more detail, particularly delayed association/avoidance (visiting areas in which another pack has recently been) of neighboring packs, and indeed to assess the responses of African wild dogs to direct and indirect (olfactory) inter‐pack encounters.

## 5 CONCLUSIONS

The rate of growth in the availability of GPS data from free‐ranging animals has not been matched by progress in the development of mathematical techniques for analysing these data. When analysing the interaction between solitary animals, the quantification of association or avoidance between territorial conspecifics would advance our understanding of animal ecology and, in the long term, the impact of changing environments. Existing forms of such tests are predicated on assumptions about the shape of the individuals’ territory and boundaries (Dunn, 1979; Macdonald et al., 1980), or the way the animals move around their territories (Fortin et al., 2005; Latombe et al., 2014; Potts et al., 2014; Vanak et al., 2013).

In this paper, a new method for detecting avoidance and association is presented. Unlike previous work, the method makes no assumption about the shape or size of the territories, nor about the way that individuals move. It relies purely on the disassociation of the individuals’ movement through permutations. The main assumption of this method is that the division of the data into blocks (e.g., days, weeks, etc.) is performed appropriately. The division must preserve patterns in the spatio‐temporal behavior of the animals. For example, it makes no sense to break the day into twelve hour periods; in this case, a block containing an habitual 2 p.m. visit to a watering hole could be paired with a 2 a.m. sleep. Likewise, where there are seasonal variations in the data, for example, on some days the watering hole is dry, on others not, the effects of seasonality must be taken into account in the permutations.

An extensive series of simulations suggest that this method has a low rate of false positives, that is, it is unlikely to suggest an association if there is none. The false positive error rate ranges from 3% to 1%. As expected, the false negative results, that is, suggesting no association when there is one, is most affected by the strength of the association. This is strongest in the value of the association time, which defines how long the individuals stay within close proximity of each other.

Among other things, this new method permits the analysis of territorial behavior in animals. Both the presence and absence of positive spatial association between individuals or groups are biologically interesting phenomena. In Section 3.2, the method was applied to data collected from GPS collars on individual leopards in which significant positive association was established between some male‐male as well as male‐female leopard dyads, and to African wild dogs, in which there was no significant dynamic interaction detected between the packs. For the leopards, two out of six male‐female dyads were more often within close proximity of each other than would be expected by chance. This is most likely related to courtship and mating, and conforms to biological expectations. Interestingly, we also showed that two out of five male‐male dyads were more often within close proximity of each other. This observation is in opposition to conclusions from previous work (Hornocker, 1970; Stander et al., 1997), but could be due to mutual evaluation, family relationships, or a range of unknown factors. None of the African wild dog packs were more or less often within close proximity of each other than would be expected by chance. It is possible that, although the movement patterns of individual packs bring neighbors into relatively close proximity, the risk and occurrence of direct encounters may be reduced by remote inter‐pack information exchange, probably via fresh scent signals in these areas.

More generally, our method for avoidance and associations could be applied to epidemiological questions. If individuals are more often within close proximity of each other than expected by chance, the transmission rate of diseases would be higher than that estimated using random movement models. The method could also be extended to include a time lag to determine whether individuals are more often in an area recently occupied by another animal than might be explained by chance. This could be important in cases of geo‐located time‐limited phenomena such as scent marking or the transmission of parasites or infectious agents through the environment.

## AUTHOR CONTRIBUTIONS

S.C., S.H., and J.S.‐T. conceived the ideas. S.C., S.H., J.S.‐T., and T.F. designed the methodology. A.B.S., N.R.J., and T.M.H. led data collection. S.C. performed the analysis and simulations and led the writing of the manuscript. All authors contributed critically to discussion of ideas, revision of the manuscript, and gave final approval for publication.

## Data Availability

The data presented in this manuscript are on *Panthera pardus* and *Lycaon pictus* which are listed as vulnerable and endangered species respectively, with some risk of poaching and illegal trade for *Panthera pardus*. Therefore the data is not made publicly available.

## ENDNOTE

1 The code used for this analysis, written in Matlab (MATLAB, 2014) can be found at https://github.com/ChisholmSarah/Avoidance.
