# Supplementary material for: Parsimonious test of dynamic interaction
Source: Ecol Evol. 2019 Feb 8;9(4):1654–64. doi: 10.1002/ece3.4805 (PMC6392374; doi:10.1002/ece3.4805)
Supplement: Supplementary file 1 [file ECE3-9-1654-s001.pdf]

1 Supplementary Material

1.1 Examples of Significant Geographic Locations

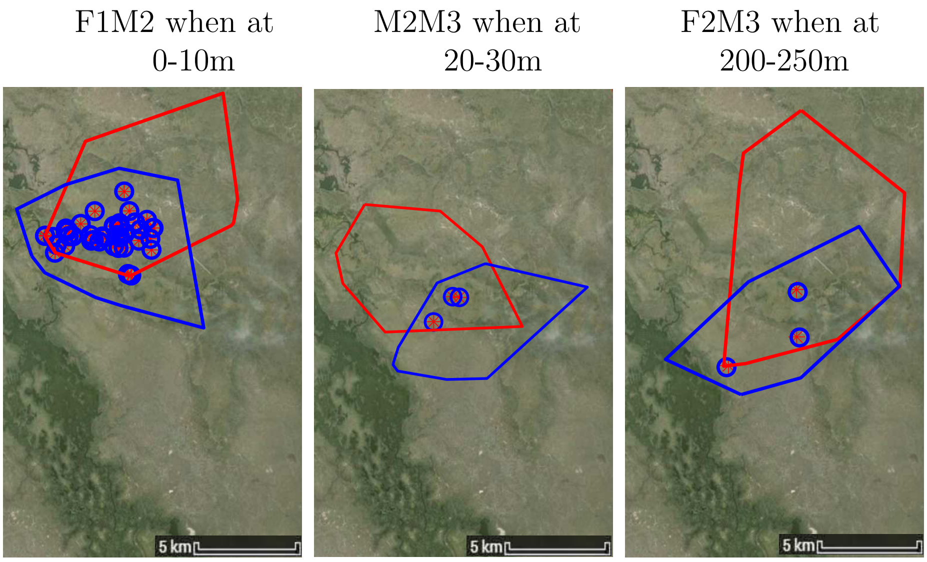

Figure 1: Representative examples of the geographic locations at which leopards are found when separated by a statistically significant distance. An estimate of the extent of each leopards territory is given using the convex hull of the full set of observations (the blue and red lines) and plotted on top of the individuals' locations (red stars and blue circles).

1.2 Time Series Plots of Distances Between the Individuals

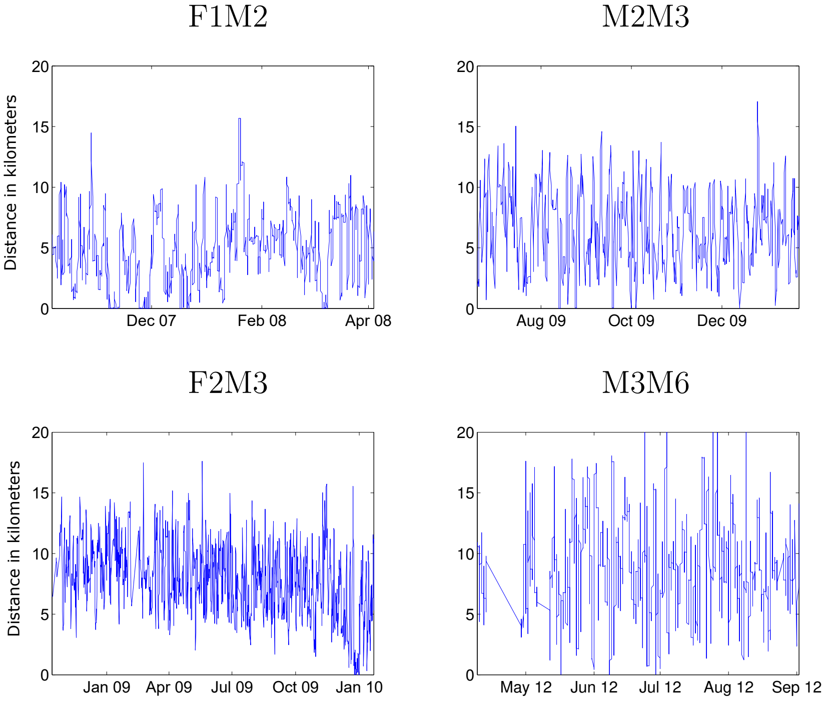

Figure 2: Representative examples of the time series plots of the leopard dyads. They show the distance between the two individuals over time.

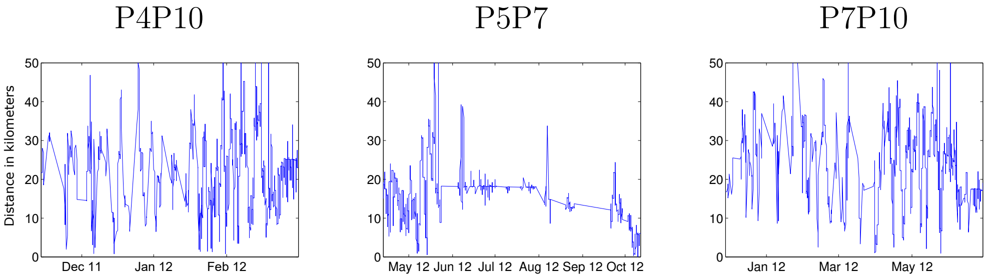

Figure 3: Representative examples of time series plots of the African wild dog packs. They show the distance between the two packs over time. The straight lines that are close to horizontal are due to the two individuals not being tagged simultaneously, in which case that period was not included in the analysis. This is particularly visible in P5P7.

1.3 Tables of Proportions of Simulations Correctly Identified as having, or not having, an Association

Association Time (in steps)	No. correctly identified as MTEpos (%)	No. correctly identified as MTEneg (%)	No. correctly identified as LTEneg (%)
1	1073/1795 (60%)	5927/6048 (98%)	7880/8064 (98%)
2	1405/1795 (78%)	5945/6048 (98%)	7725/8064 (96%)
3	1513/1795 (84%)	5951/6048 (98%)	7572/8064 (94%)
4	1601/1795 (89%)	5962/6048 (99%)	7440/8064 (92%)
5	1658/1795 (92%)	5976/6048 (99%)	7323/8064 (91%)

Table 1: Effect of Association Time: Details of the number of simulations correctly classified as being more (MTEpos) often, not being more (MTEneg) often, and not being less (LTEneg) often within the distances tested than expected by chance. The results are divided into the number of time steps (first column) the individuals spent in the association distance of each other. In each column the number before the forward-slash is the number of correctly classified distances, the number after the forward-slash is the number of distances that should be flagged, and the number in brackets is the proportion of correctly identified distances.

Sensing Distance (in meters)	No. correctly identified as MTEpos (%)	No. correctly identified as MTEneg (%)	No. correctly identified as LTEneg (%)
250	1046/1410 (74%)	4996/5040 (99%)	6432/6720 (96%)
300	1116/1465 (76%)	4976/5040 (99%)	6430/6720 (96%)
350	1173/1495 (78%)	4949/5040 (98%)	6362/6720 (95%)
400	1248/1510 (83%)	4923/5040 (98%)	6290/6720 (94%)
450	1350/1540 (88%)	4965/5040 (99%)	6234/6720 (93%)
500	1317/1555 (85%)	4952/5040 (98%)	6192/6720 (92%)

Table 2: Effect of Sensing Distance: Details of the number of simulations correctly classified as being more (MTEpos) often, not being more (MTEneg) often, and not being less often (LTEneg) within the distances tested than expected by chance. The results are divided into the sensing distance (first column). In each column the number before the forward-slash is the number of correctly classified distances, the number after the forward-slash is the number of distances that should be flagged, and the number in brackets is the proportion of correctly identified distances.

Observation Length (in days)	No. correctly identified as MTEpos (%)	No. correctly identified as MTEneg (%)	No. correctly identified as LTEneg (%)
100	749/1080 (69%)	4982/5040 (99%)	6543/6720 (96%)
150	1121/1465 (77%)	4933/5040 (98%)	6414/6720 (95%)
200	1252/1590 (79%)	4949/5040 (98%)	6275/6720 (93%)
250	1298/1555 (83%)	4961/5040 (98%)	6253/6720 (93%)
300	1415/1665 (85%)	4969/5040 (99%)	6249/6720 (93%)
350	1415/1620 (87%)	4967/5040 (99%)	6206/6720 (92%)

Table 3: Effect of Observation Length: Details of the number of simulations correctly classified as being more (MTEpos) often, not being more (MTEneg) often, and not being less (LTEneg) often within the distances tested than expected by chance. The results are divided into the observation length (first column). In each column the number before the forward-slash is the number of correctly classified distances, the number after the forward-slash is the number of distances that should be flagged, and the number in brackets is the proportion of correctly identified distances.

Observation Length (in days)	No. correctly identified as NTEneg (%)	No. correctly identified as LTEneg (%)
100	6670/6720 (99%)	6685/6720 (99%)
150	6535/6720 (98%)	6615/6720 (98%)
200	6585/6720 (98%)	6680/6720 (99%)
250	6560/6720 (98%)	6645/6720 (99%)
300	6550/6720 (97%)	6670/6720 (99%)
350	6555/6720 (98%)	6620/6720 (99%)

Table 4: Effect of Observation Length when there is no association: Details of the number of simulations correctly classified as not being less (LTEneg) or more (MTEneg) often within the distances tested than expected by chance in the no association scenario. The results are divided into the observation length (first column). In each column the number before the forward-slash is the number of correctly classified distances, the number after the forward-slash is the number of distances that should be flagged, and the number in brackets is the proportion of correctly identified distances.
